# Targeted Colostrum Bioactive Proteins Improve Gastrointestinal Symptoms and Modulate the Gut Microbiome: A Randomised Double-Blind Placebo-Controlled Trial

**DOI:** 10.3390/nu18213561

**Published:** 2026-10-28

**Authors:** Amanda Rao, Tzippora Shalem, Romeo Batacan, Yadav Sharma Bajagai, David Briskey

**Affiliations:** 1 RDC Clinical, Brisbane 4006, Australia; david@rdcglobal.com.au; 2 Sackler Faculty of Medicine, Tel-Aviv University, Tel Aviv 6997801, Israel; 3 Shamir Medical Center, Zerifin 7033001, Israel; 4 School of Health, Medical and Applied Sciences, Central Queensland University, Rockhampton 4702, Australia

**Keywords:** upper gastrointestinal symptoms, bovine colostrum, MaolactinGI™, gut microbiome, abdominal discomfort

## Abstract

Background: Gastrointestinal (GI) symptoms such as indigestion, bloating and reflux are common and can negatively affect quality of life. Bovine colostrum is a rich source of nutrients and other bioactive constituents that has been shown to be beneficial for gut health. Methods: This 12-week, randomised, double-blind, placebo-controlled, 3-arm study aimed to evaluate the effects of MaolactinGI™, a proprietary bovine colostrum supplement, on upper GI symptoms and gut microbiome composition in healthy adults aged 18 years and older experiencing moderate GI discomfort. Participants completed a 4-week run-in period followed by an 8-week intervention period. Outcomes measured included changes in upper GI symptoms via the Gastrointestinal Symptom Rating Scale (GSRS), Gastroesophageal Reflux Disease Questionnaire (GerdQ), Bloating Symptoms Visual Analogue Scale (BSVAS), Digestion-associated Quality of Life Questionnaire (DQLQ), Bristol Stool Chart, intestinal permeability, faecal calprotectin, serum inflammatory markers, gut microbiome analyses, blood safety markers and adverse events. Results: Ninety eligible participants were allocated to either the high-dose (500 mg/day), low-dose (250 mg/day), or placebo groups, and data from 83 participants were analysed. The high-dose MaolactinGI™ group demonstrated statistically significant improvements in the total GSRS score and DQLQ score compared with placebo, suggesting a meaningful reduction in total symptom burden and improved quality of life. The improvements in GSRS were clinically meaningful in size, supporting the practical relevance of MaolactinGI™ for adults experiencing everyday upper GI discomfort. High-dose supplementation also preserved microbial richness relative to placebo (Chao1); exploratory within-group analyses further indicated enrichment of SCFA-associated commensal taxa. The intervention was well tolerated and demonstrated an acceptable safety profile. Conclusions: These findings support the potential of targeted colostrum-derived bioactive protein fractions as a novel nutritional strategy for supporting GI health and microbiome balance.

## 1. Introduction

Many individuals experience a variety of gastrointestinal (GI) symptoms from time to time, such as abdominal discomfort, dyspepsia, bloating, and reflux [1]. GI symptoms are highly prevalent in otherwise healthy populations and can result in significant global health care costs and impaired health-related quality of life (QOL) [2]. Epidemiological studies estimate that more than 40% of the global population experiences functional GI disorders and up to 68–80% of adults report experiencing at least one mild to moderate GI symptom on a regular basis [3,4,5,6,7]. These symptoms may arise from functional GI disturbances, mild mucosal inflammation, alterations in gut barrier integrity, or dysbiosis of the intestinal microbiome [1,8].

The GI tract represents a complex physiological system responsible not only for nutrient digestion and absorption but also for immune regulation and host–microbiome interactions [9]. Disruptions in this ecosystem may lead to functional GI symptoms, including dyspepsia, abdominal pain, early satiety, nausea, and reflux. Even in individuals without diagnosed GI disease, these symptoms can substantially impair digestive well-being and daily functioning [7].

Bovine colostrum is the first milk produced by mammals following parturition and contains a rich array of bioactive molecules that contribute to immune protection, tissue development, and microbial regulation. Bioactive components found within bovine colostrum include immunoglobulins, glycoproteins, antimicrobial proteins, and structural proteins involved in epithelial protection and immune signalling. These bioactive molecules have been investigated in several clinical contexts related to GI health, including inflammatory bowel disease, infectious diarrhoea, and mucosal injury [10,11,12,13,14,15,16].

Unlike conventional probiotic or prebiotic interventions that attempt to directly modify microbial populations, bioactive protein fractions derived from colostrum may influence the gut ecosystem through host-directed mechanisms. These proteins interact with epithelial cells and mucosal immune pathways that regulate barrier integrity and microbial homeostasis [13,14,15,17]. By modulating the intestinal environment, bioactive proteins may indirectly influence the composition and function of the gut microbiome.

Traditional bovine colostrum supplements are typically delivered as bulk powders containing a broad mixture of proteins, lipids, lactose, and casein. MaolactinGI™ is a colostrum-derived fraction manufactured using a multi-step filtration and purification process designed to reduce casein, lipids, and low-molecular-weight components while concentrating a fraction enriched in proteins such as immunoglobulins, glycoproteins, and other structural proteins. These components are proposed to interact with mucosal and immune signaling pathways within the GI tract. Based on these mechanisms, the present study aimed to evaluate the effects of MaolactinGI™ supplementation on upper GI symptoms, digestive-related quality of life, and gut microbiome composition in adults experiencing moderate GI discomfort.

## 2. Materials and Methods

### 2.1. Trial Design and Setting

This randomised, double-blind, placebo-controlled, 3-arm study was of 12 weeks’ duration, with a 4-week run-in period as the control phase and an 8-week intervention period. It was conducted at a research site (RDC Clinical) in Brisbane, Australia. Participants were also recruited remotely from Sydney, Australia.

### 2.2. Ethics and Trial Registration

The study was conducted in accordance with the Declaration of Helsinki and the International Council for Harmonisation Good Clinical Practice (ICH-GCP) guidelines. The protocol and all study procedures were approved by the Bellberry Human Research Ethics Committee (Approval No. 2023-06-749). Written informed consent was obtained from all participants prior to enrolment. The trial was registered at ClinicalTrials.gov (Identifier: NCT06104917).

### 2.3. Study Population

Generally healthy adults aged 18 years and over, with a body mass index (BMI) < 35 kg/m^2^, with stable dietary habits, experiencing moderate GI symptoms (1 or more of the following symptoms at least once a week for more than 3 months: reflux, heartburn, regurgitation, nausea, bloating or abdominal pain) were recruited. Participants with any serious chronic illness, including cancer, cardiovascular diseases, diabetes, and autoimmune and inflammatory conditions, were excluded. Pregnant or nursing women were also excluded (see Supplementary Table S1 for full inclusion/exclusion criteria).

### 2.4. Interventions

The three arms in this study were: the low-dose active arm, which received 1 capsule containing 250 mg MaolactinGI™ and 1 capsule of 250 mg maltodextrin daily; the high-dose active arm who received 2 capsules MaolactinGI™ daily (a total of 500 mg MaolactinGI™); and the placebo arm, who received 2 capsules containing maltodextrin only (a total of 500 mg maltodextrin a day). Participants were instructed to take 2 capsules orally, once daily, before the morning meal. The low-dose active group received one active and one placebo capsule to ensure identical capsule counts across arms. The randomisation sequence was generated using simple randomisation (www.sealedenvelope.com) (accessed on 8 February 2024) in a 1:1:1 allocation ratio, by an independent person not involved in the conduct of the trial. The randomisation code was generated prior to trial commencement, with access restricted to those not involved in participant care or outcome assessment. All investigational products were packed in identical containers and labelled identically except for a unique product number corresponding to the randomisation code. The placebo capsules were identical in size, colour and consistency to the active treatment capsules. The investigators, trial participants and the statistician performing the data analysis remained blinded to product allocation throughout the trial. Both active and placebo products were manufactured in accordance with Good Manufacturing Practice (GMP) standards. Participants were monitored for dosing compliance at each study follow-up. At the completion of the trial, each participant was asked to return any residual capsules.

### 2.5. Outcomes

The primary outcome was the change in upper GI symptoms as evaluated using the Gastrointestinal Symptom Rating Scale (GSRS), defined a priori as the change from baseline in GSRS total score at Week 8. Secondary outcomes included changes in: upper GI symptoms such as dyspepsia and reflux via the Gastroesophageal Reflux Disease Questionnaire (GerdQ) and Bloating Symptoms Visual Analogue Scale (BSVAS); stool frequency and consistency via the Bristol Stool Chart Questionnaire; intestinal permeability via plasma zonulin and a 6-h urine test; gut inflammation via faecal calprotectin from stool sample; quality of life (QoL) via the Digestion-associated Quality of Life Questionnaire (DQLQ); diet via the 24-h Dietary Recall; inflammatory markers (tumour necrosis factor [TNF]-α, interleukin [IL]-1β, IL-6, and IL-8, C-reactive protein [CRP], nuclear factor-kappa B [Nf-Kb]) from fasted blood test; gut microbiome analyses; and safety via adverse events and general pathology safety markers (electrolyte and liver function test [E/LFT], cholesterol and glucose).

### 2.6. Study Questionnaires

The GSRS contains 15 items, each rated on a seven-point Likert scale, from “no discomfort at all” to “very severe discomfort”; a reduction in scores represents improvement in the severity of symptoms. The GSRS is validated to evaluate symptoms in the following five domains: abdominal pain, reflux symptoms, diarrhoea, indigestion, constipation, and total GSRS [18]. The DQLQ is a valid and reliable self-reported questionnaire that reflects the sum of 9 statements scored on a 10-point Likert scale (0 = never to 1 = always) and provides a total DQLQ score that ranges from 0–9, with a higher score indicating poorer quality of life [19]. GerdQ is a useful tool in the diagnosis of gastroesophageal reflux disease (GERD). It consists of six questions with positive and negative predictors. A total GerdQ score of >8 is used to diagnose GERD [20]. The BSVAS consists of three questions related to bloating that are scored from 0–10, with 0 being no symptoms and 10 being most severe. The symptoms rated include “stomach feeling full or uncomfortable”, “stomach distension” and “the skin around your stomach feeling stretched or tight”. The Bristol Stool Chart is a validated tool used to classify the stool into 7 categories based on shape and consistency [21].

### 2.7. Study Procedures

Once enrolled, on Day 1, participants completed the GSRS, GerdQ, BSVAS and DQLQ questionnaires, the Bristol Stool Chart, and the 24-h dietary recall. This was followed by a 4-week (28-day) run-in period without any intervention. Prior to the intervention period baseline (Day 28) appointment, participants collected a urine and stool sample. At the baseline appointment, participants’ height, weight, hip and waist circumference, and vital signs were measured, and study questionnaires were completed. A fasted blood sample was obtained. Study questionnaires were completed at weeks 2, 4 and 8. Participants completed the dietary recall and collected another urine and stool sample prior to their week 8 (Day 84) appointment, where the baseline measures were repeated. Adverse events and compliance were monitored at each point of contact.

### 2.8. Statistical Analysis

The sample size calculation was performed a priori using G*Power (version 3.1). Assuming a clinically meaningful 20% reduction in the primary outcome (GSRS), with an alpha error probability of 0.05, power of 0.8. With an effect size of 0.7, a minimum of 26 participants per group was required. The analysis population was a modified intention-to-treat (mITT) population, defined as all randomised participants who received the allocated study product. Missing data were imputed using last observation carried forward (LOCF), whereby a missing value was replaced with the participant’s preceding observed value; where multiple consecutive timepoints were missing, the last observed value was carried forward across all missing timepoints. Continuous variables were summarised as mean ± standard deviation (SD) for change from baseline at each post-baseline timepoint (Weeks 2, 4, and 8). The primary efficacy endpoint was defined a priori as the change from baseline in GSRS total score at Week 8. Multiplicity was addressed at the design stage by pre-specifying this single primary endpoint. Between-group comparisons of the change from baseline scores (rather than the raw baseline and follow-up scores) at each timepoint were performed independently using two-sided independent samples *t*-tests, with each comparison made between two groups (high-dose vs. placebo, low-dose vs. placebo, and high-dose vs. low-dose). Each timepoint for the clinical outcomes was analysed as a separate cross-sectional assessment of change from baseline; no longitudinal or repeated measures modelling was undertaken for these outcomes. Normality was assessed using the Shapiro–Wilk test, and homogeneity of variance was evaluated using Levene’s test. Independent-samples *t*-tests were applied where the assumptions of normality and equal variances were satisfied; Welch’s *t*-test was used where the assumption of equal variances was not met. Statistical analysis was conducted using IBM SPSS Statistics (version 31.0.1.0 or later).

Microbiome outcomes were additionally evaluated using longitudinal models incorporating group, time, and the group × time interaction to compare changes in each active treatment group directly with changes in the placebo group. Alpha-diversity outcomes included Shannon diversity, Chao1 richness, and observed richness. Genus-level differential abundance was assessed using LinDA, with Benjamini–Hochberg correction for multiple comparisons, and ANCOM-BC2 was performed as a sensitivity analysis. Differences in overall microbial community composition were assessed using PERMANOVA.

Analyses at Weeks 2 and 4 and domain-level GSRS comparisons were considered secondary/exploratory and were not corrected for multiple comparisons. All statistical tests were two-sided with a nominal significance level of α = 0.05.

### 2.9. Stool Sample Analysis

Faecal sample collection was conducted by the participant in the privacy of their home and returned to the clinic within 7 days of collection. Participants were provided with a faecal collection kit (OMR-200; DNA Genotek, Ottawa, ON, Canada) and instructions during their first clinic visit. Once returned to the clinic, faecal samples were stored at −80 °C until analysis. Faecal samples were analysed for microbiome quantification and calprotectin.

#### 16S rRNA Gene Amplicon Sequencing, Bioinformatics and Statistics

Total DNA from faecal samples was isolated using the DNA mini spin columns (Enzymax LLC, Lexington, KY, USA). DNA concentration and purity were measured using a NanoDrop One spectrophotometer (Thermo Fisher Scientific, Wilmington, DE, USA). The V3-V4 hypervariable region of the 16S rRNA gene was amplified using the primer pair 341F (5′-CCTACGGGNGGCWGCAG-3′) and 785R (5′-GACTACHVGGGTATCTAATCC-3′) and sequenced on the Illumina MiSeq platform. Raw FASTQ file integrity was verified using MD5 checksums, and sequence quality was assessed using FastQC v0.12.1 [22]. Reads were processed in QIIME2 (v2024.10) [23] using the DADA2 plugin (v1.30.0) [24] for quality filtering, denoising, and chimera removal. Taxonomy was assigned to the resulting ASVs using a Naïve Bayes classifier [25] trained on the SILVA 138 database [26] at 99% sequence similarity.

All downstream analyses were conducted in R (v4.4.2). Sequences were rarefied to account for unequal sequence depth before calculating alpha diversity and beta diversity. A total of 150 faecal samples were received for analysis (76 baseline and 74 final [Week 8]), of which 73 were paired baseline and final samples from the same participant. The mITT approach was not applied in the absence of a baseline sample; microbiome analyses were therefore conducted on the 76 participants with a baseline sample. Samples were classified into three treatment groups (low dose, high dose, and placebo) and two time points (Baseline and Final), with repeated measurements linked by participant ID. The primary longitudinal estimand throughout the analysis was the Treatment × Time interaction, representing whether the Baseline-to-Final change in either treatment group differed from that observed in the placebo group. Alpha-diversity outcomes were analysed using linear mixed-effects models implemented with lme4/lmerTest, with Treatment, Time and Treatment × Time as fixed effects and participant as a random intercept. Satterthwaite approximations were used for inference on fixed effects. Treatment-versus-placebo difference-in-differences contrasts and 95% confidence intervals were estimated using emmeans [27,28]. Complete-pair ANCOVA models comparing Final values between groups while adjusting for Baseline values were performed as sensitivity analyses. Treatment effects on community composition were evaluated using PERMANOVA in the vegan package with the model Treatment × Time; permutations were restricted within participants to account for repeated measurements, using 9999 permutations [29,30]. Treatment-specific interaction tests compared each active treatment directly with placebo. Homogeneity of multivariate dispersion was assessed using PERMDISP (betadisper) [31], and principal coordinate analysis (PCoA) was used for graphical representation of longitudinal community trajectories.

Differential-abundance analyses were conducted on unrarefied genus-level counts. Samples with fewer than 1000 reads were excluded, and genera were retained if present in at least 10% of samples and detected at both time points in at least three participants. Differential longitudinal changes were assessed independently using ANCOM-BC2 and LinDA, with models containing Treatment, Time, and Treatment × Time and a participant random effect. ANCOM-BC2 accounts for sampling-fraction and compositional biases and supports repeated-measures designs [32], whereas LinDA (linear models for differential abundance) applies bias-corrected linear models to centred log-ratio-transformed microbiome data and supports mixed-effects models [33]. Multiple testing was controlled using the Benjamini-Hochberg false-discovery rate (FDR) procedure. Linear discriminant analysis effect size (LEfSe) was used for exploratory, within-arm comparisons of Baseline and Final samples to identify taxa that discriminated between time points. These analyses did not test whether changes differed between treatment groups. Between-group treatment effects were assessed separately using the longitudinal LinDA models with a Treatment × Time interaction, with ANCOM-BC2 used as a sensitivity analysis.

### 2.10. Faecal Calprotectin

Faecal calprotectin samples were dehydrated using a centrifugal vacuum dryer and dry weights calculated. The samples were reconstituted in assay buffer and calprotectin measured as per instructions using an Epitope Diagnostics Calprotectin ELISA kit. The plates were read on a Biobase BL-EK-10C plate reader as per the kit manufacturer’s recommendations.

## 3. Results

**Figure 1:**
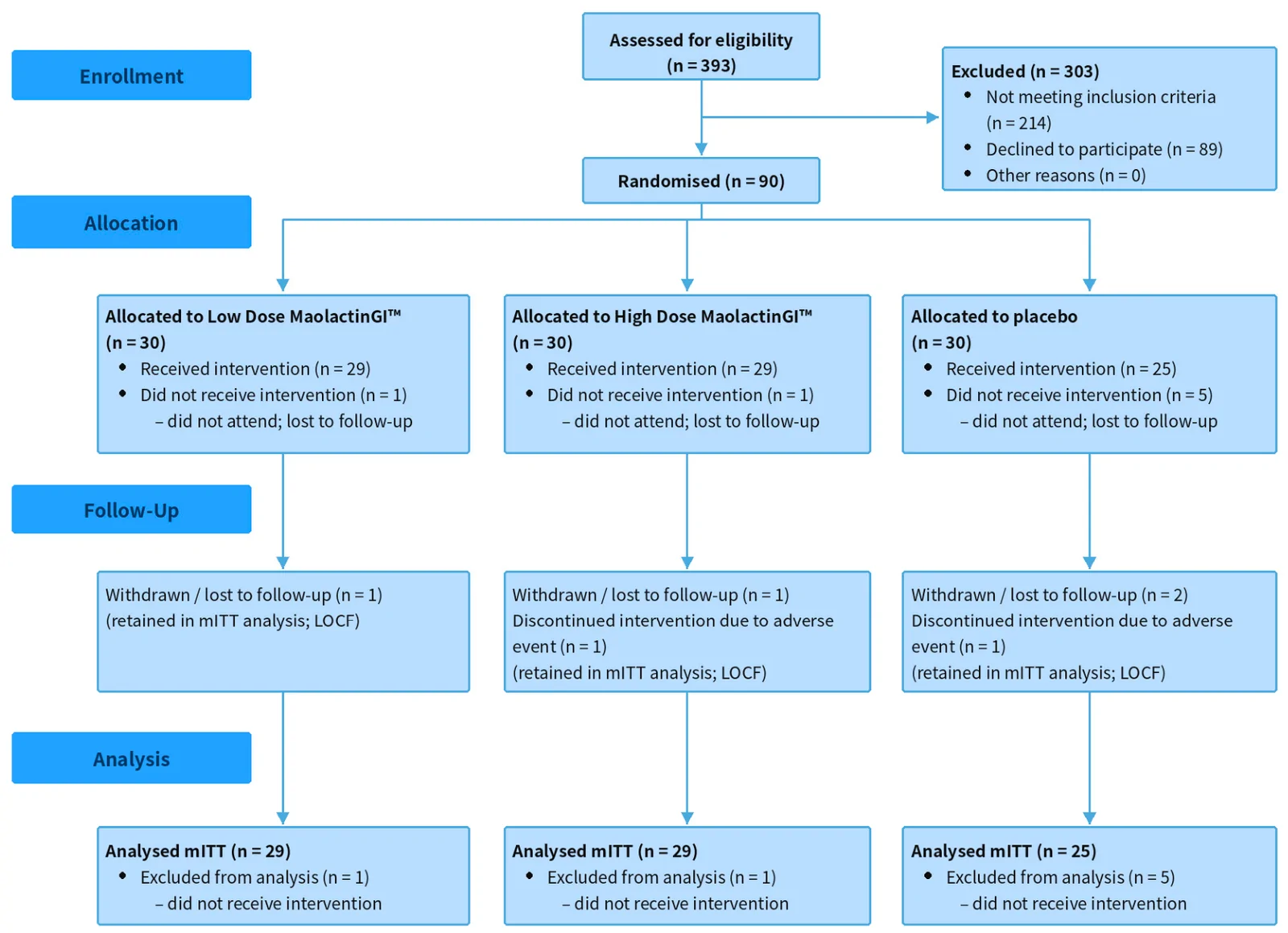
Consort Flow Chart.

Participants were recruited from both Brisbane and Sydney, Australia. Enrolment took place from 21 February 2024 to 28 May 2025. Ninety eligible participants were enrolled and allocated to one of the three study groups, and data from 83 participants were analysed using modified intention-to-treat (mITT). Seven randomised participants (low-dose *n* = 1, high-dose *n* = 1, placebo *n* = 5) did not attend the clinic to collect study product and commence the study and provided no reason; these participants were lost to follow-up before receiving any study product and were therefore not included in the mITT population. The imbalance across groups arose by chance, with all seven losses occurring after randomisation but before any exposure to study product. The six participants who withdrew after commencing the intervention (low-dose *n* = 1, high-dose *n* = 2, placebo *n* = 3) were retained in the mITT population, with missing data handled using LOCF. Figure 1 describes the flow of participants through the trial.

### 3.1. Study Population

The baseline characteristics of participants are shown in Table 1. The three groups were well matched, with minor variances in baseline values. The participant characteristics comparing baseline and week 8 are presented in Supplementary Table S2.

### 3.2. Primary Outcome—Gastrointestinal Symptom Rating Scale (GSRS)

**Figure 2:**
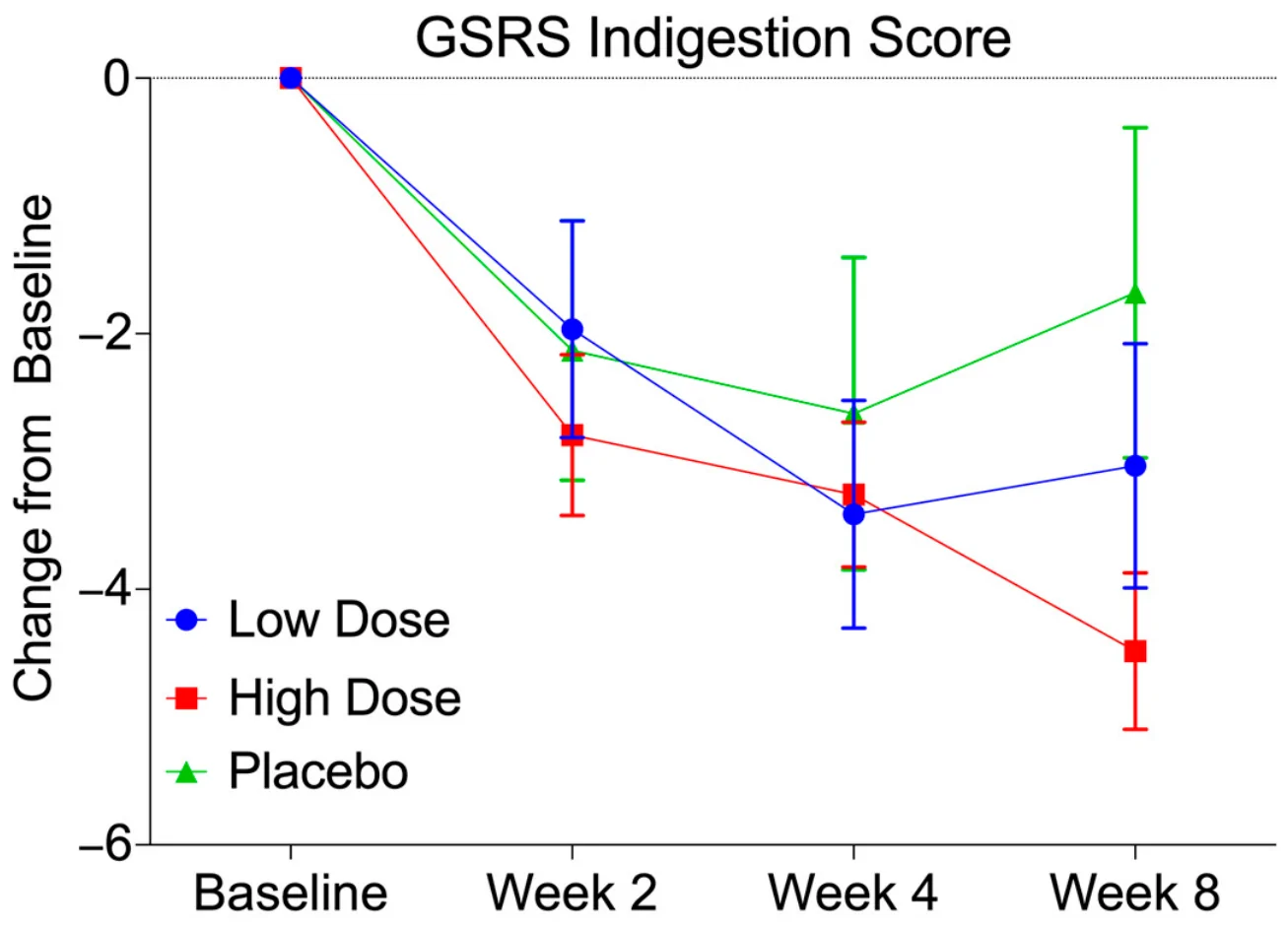
Change from Baseline in GSRS Indigestion Score.

At baseline, all groups showed similar baseline GSRS scores across domains, indicating comparable symptom severity at study initiation (Table 2). Both active treatment groups demonstrated improvements in GI symptoms over the 8-week study period, with the high-dose group showing more pronounced and statistically significant effects compared to placebo. The high-dose treatment achieved statistically significant improvements compared to placebo in the change in symptoms from baseline to week 8 in three key domains: abdominal pain (high-dose −2.76 ± 2.36 vs. placebo −1.12 ± 2.39; *p* = 0.036), indigestion (high-dose −4.48 ± 3.30 vs. placebo −1.68 ± 6.59; *p* = 0.045) (Figure 2), and constipation (high-dose −2.48 ± 2.44 vs. placebo −0.40 ± 4.57; *p* = 0.035).

**Figure 3:**
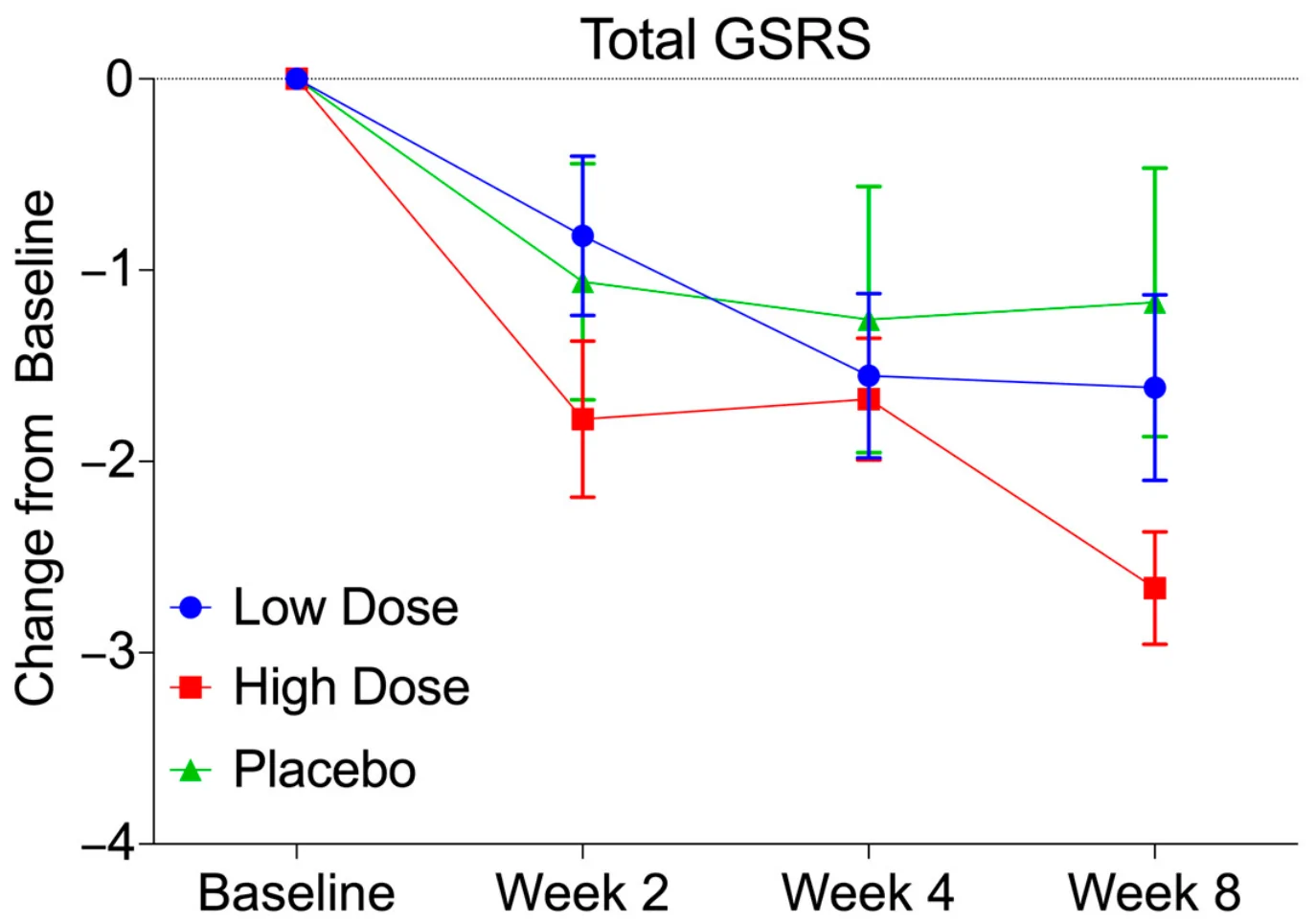
Change from Baseline in Total GSRS.

The change in the total GSRS score for the high-dose group was statistically significant compared to placebo (high-dose −2.66 ± 1.58 vs. placebo −1.17 ± 3.58; *p* = 0.044) (Figure 3). The high-dose group also outperformed the low-dose group in the diarrhoea domain (−1.62 ± 3.18 vs. 0.14 ± 3.42; *p* = 0.047). No other significant differences were observed between the high-dose and low-dose groups for the total GSRS or any other GSRS domain. While the low-dose group showed numerical improvements in most domains, these changes did not reach statistical significance compared to placebo.

### 3.3. Gastrointestinal Questionnaire Outcomes

All three groups showed improvements in overall quality of life (DQLQ) over time (Table 3). The *t*-test showed that the high-dose group demonstrated significant improvements in the change from baseline in Total DQLQ Scores compared to placebo at week 2 (−1.24 ± 1.40 vs. placebo 0.18 ± 1.56; *p* = 0.001), week 4 (−1.30 ± 1.29 vs. placebo −0.47 ± 1.36; *p* = 0.035), and week 8 (−1.55 ± 1.18 vs. placebo −0.53 ± 1.62; *p* = 0.013), indicating sustained enhancement in digestive-related quality of life. The high-dose group also outperformed the low-dose group at week 8 (high-dose −1.55 ± 1.18, low-dose −0.53 ± 1.61; *p* = 0.02).

**Figure 4:**
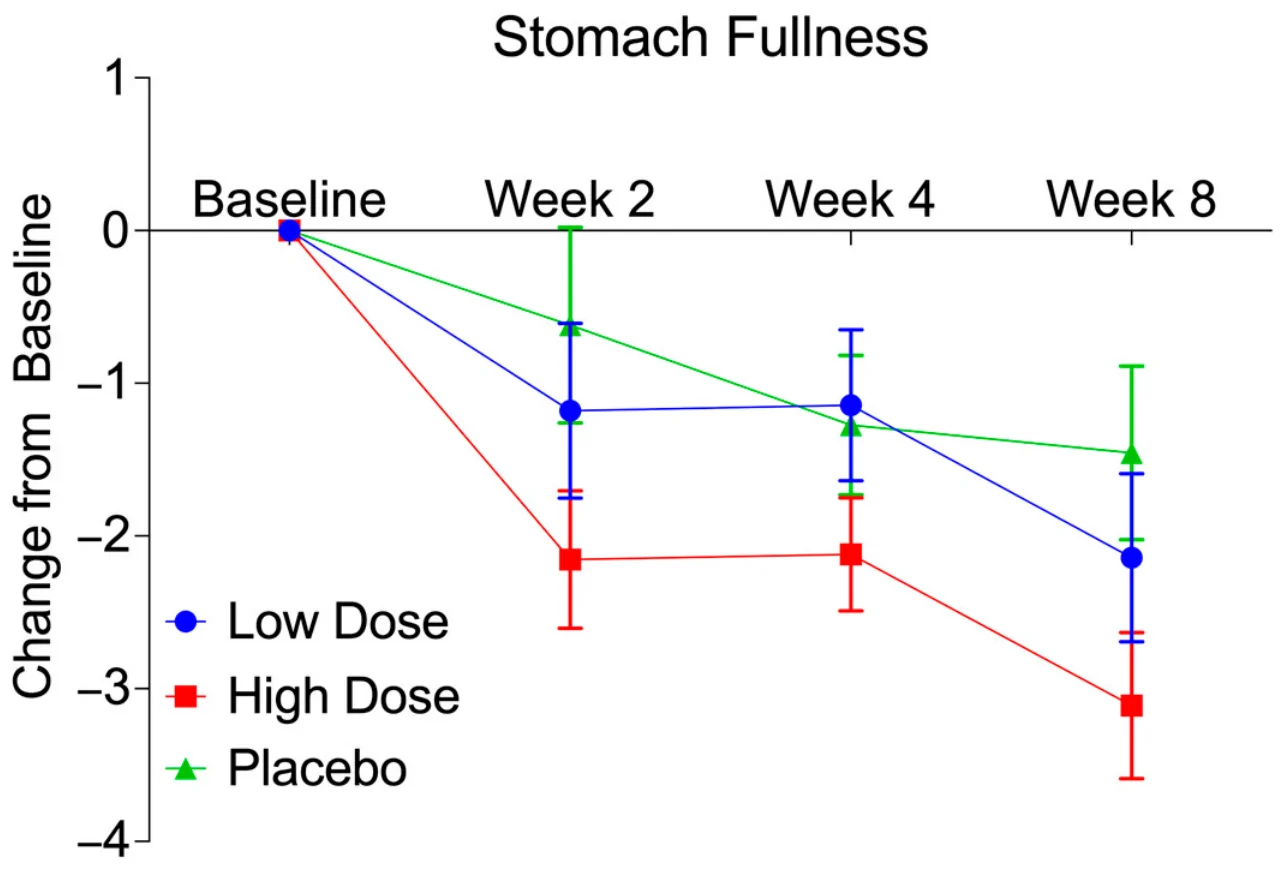
Stomach Feeling Full VAS score.

Review of dietary recall indicated no changes in diet over the study in any of the groups. With regard to gastroesophageal reflux, total GERD-Q scores remained relatively stable across all groups throughout the study, with no significant differences observed between treatment groups and placebo (Table 3). All three groups had an improvement in bloating symptoms (BSVAS) over the 8-week period (Table 3). The high-dose group showed a significant improvement in the change in “stomach feeling full” symptoms between baseline and week 8 compared to placebo (high-dose group −3.11 ± 2.48 vs. −1.45 ± 2.66 for placebo; *p* = 0.029) (Figure 4). There were no other significant differences found between groups. The Bristol Stool Shape scores and number of bowel movements remained stable across all groups, with no significant differences found between groups (Table 3).

### 3.4. Calprotectin and Inflammatory Markers

There were no statistically significant changes in blood inflammatory markers between groups or time points. Faecal calprotectin levels increased slightly in the high-dose group between baseline and week 8 (+1.17 ug/g ± 6.07), however, this change was not significant (Supplementary Tables S3 and S4).

### 3.5. Intestinal Permeability

Intestinal permeability was measured by the lactulose-mannitol intestinal permeability test, or dual-sugar test. The results showed participants in the study had a low lactulose:mannitol ratio, suggesting they had normal permeability at baseline, and this did not change during the study (*p* > 0.05) (Supplementary Table S5).

### 3.6. Gut Microbiome

#### 3.6.1. Alpha Diversity (Richness and Shannon Index)

**Figure 5:**
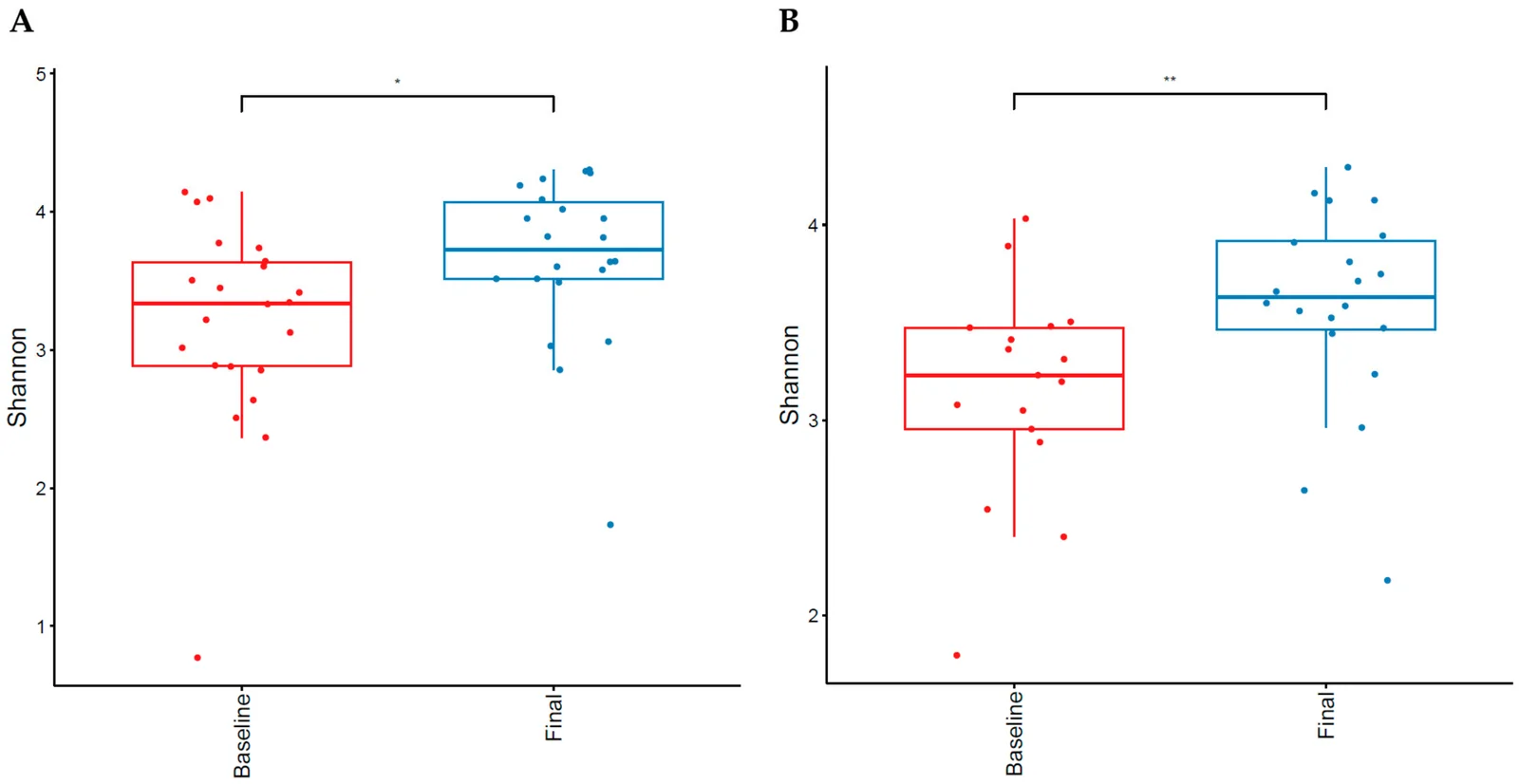
Microbial diversity (Shannon index) in the high-dose Maolactin™ group (**A**) vs. Placebo group (**B**) at Baseline and final time points. Box plots show the median (horizontal line), interquartile range (box), and range (whiskers), with individual participant values shown as dots. Red = Baseline; Blue = Final. In both groups, the change was statistically significant. *p*-values reflect within-group comparisons (Baseline vs. Final). * = *p* < 0.05, ** = *p* < 0.01.

The high-dose Group demonstrated a significant increase in microbial diversity, as measured by the Shannon index, from baseline to week 8 (within-group, Baseline vs. Final; *p* < 0.05) (Figure 5). A significant increase in Shannon diversity was also observed in the placebo group (*p* < 0.01) (Figure 5). Between-group comparisons are presented in Section 3.6.3. No consistent changes were observed in overall alpha diversity metrics in the low-dose group.

#### 3.6.2. Within-Group Taxonomic Shifts (Exploratory LEfSe Analysis)

**Figure 6:**
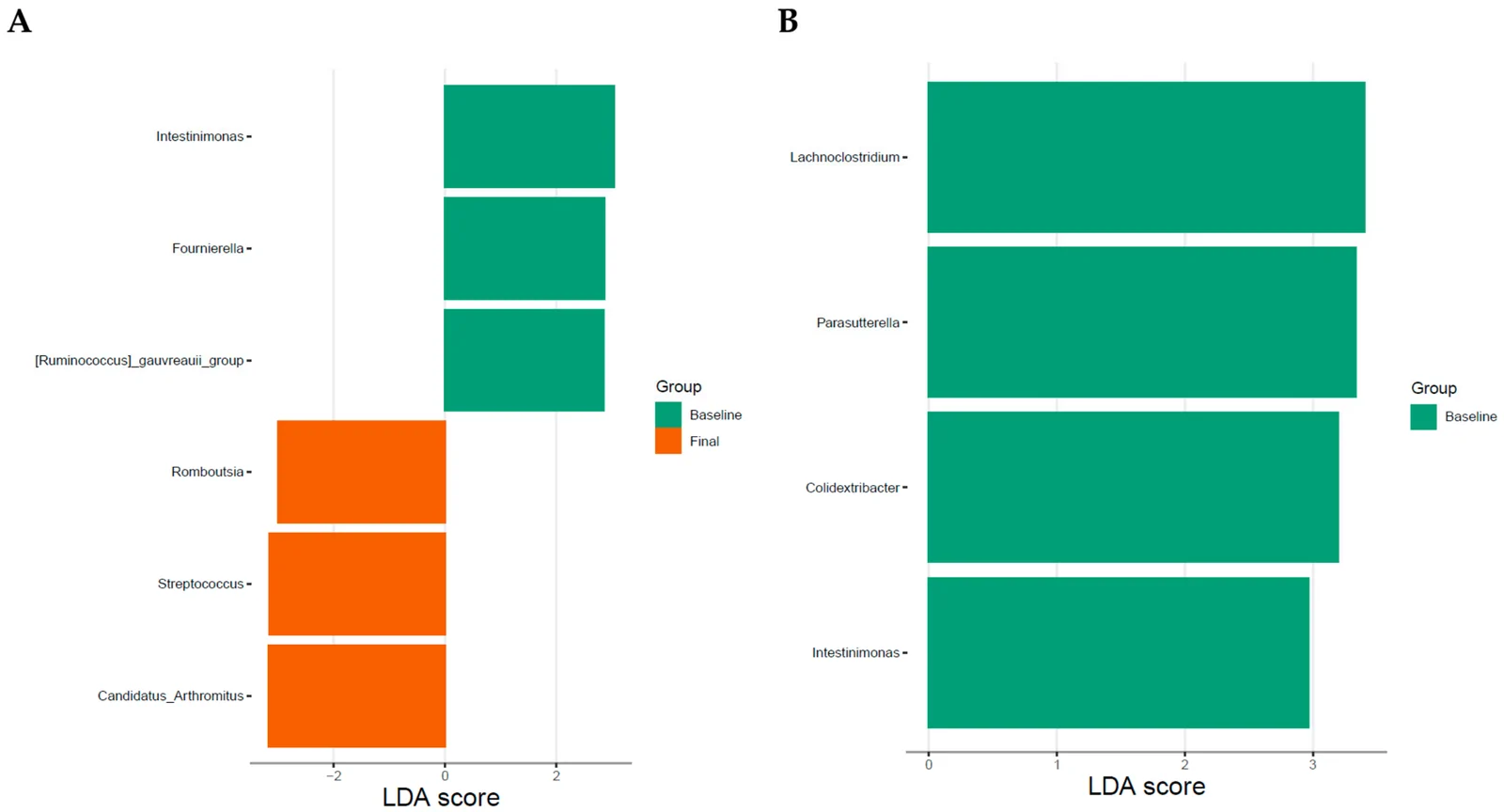
Linear discriminant analysis effect size (LEfSe) results identifying bacterial biomarkers at the genus level in the high-dose Maolactin™ group (**A**) vs. placebo (**B**). Horizontal bars represent the Linear Discriminant Analysis (LDA) scores for taxa that were significantly more abundant at Baseline (green, positive values) or Final (orange, negative values). Higher absolute LDA scores indicate greater effect size and stronger association with the respective group. The high-dose group showed enrichment of bacteria (such as *Veillonella* and *Megasphaera*) associated with the production of short-chain fatty acids (SCFAs).

The following within-group comparisons were exploratory and do not test whether changes differed from placebo. Overall, the placebo group exhibited minor temporal changes. In the low-dose group, Final samples showed a compositional shift away from opportunistic or pro-inflammatory bacteria toward a potentially more beneficial microbial profile, despite no change in overall diversity metrics. In the high-dose group, Final samples were characterized by significant increases in lactate-utilizing bacteria, specifically *Veillonella* and *Megasphaera*, as well as an abundance of *Romboutsia*, *Streptococcus*, and *Candidatus Arthromitus* (Figure 6). Both active dosing groups showed a decline in the relative abundance of several taxa typically linked to GI disturbance or inflammatory potential (e.g., *Campylobacter*, *Tyzzerella*, *Methanobrevibacter*), while many of the taxa enriched post-supplementation are associated with short-chain fatty acid (SCFA) production and carbohydrate fermentation, processes known to support gut barrier integrity.

#### 3.6.3. Between-Group Comparisons

Direct comparison of microbiome changes between the treatment and placebo groups indicated a high-dose effect on microbial richness. In the log-transformed Chao1 mixed model, the overall group × time interaction approached statistical significance $\left[F_{2,54.6},=3.03\;p=0.056\right]$. The prespecified high-dose-versus-placebo difference-in-differences contrast was statistically significant after adjustment for multiple comparisons (estimate =0.352log units, 95% CI 0.001–0.703; adjusted p=0.0496). Chao1 richness decreased in the placebo and low-dose groups but remained stable in the high-dose group, corresponding to an approximately 1.42-fold higher Final/Baseline richness ratio in the high-dose group relative to placebo. Observed richness demonstrated a similar pattern, with an estimated high-dose-versus-placebo difference of approximately 28 taxa, although this comparison did not reach statistical significance after multiplicity adjustment (p=0.0658).

At the genus level, LinDA identified a significant high-dose × time interaction for *Parasutterella* relative to placebo (log2 fold-change =3.26, adjusted p=0.0368). A positive interaction was also observed for *Bilophila* (log2 fold-change =3.37). These taxa-level findings were not replicated by ANCOM-BC2 and should therefore be interpreted as exploratory. PERMANOVA did not identify significant group × time interactions for overall community composition, indicating that the observed effects were more apparent for selected richness and taxonomic outcomes than for global microbiome structure.

### 3.7. Safety Pathology and Adverse Events

There were no significant differences between groups at baseline or week 8 for any safety pathology and metabolic function markers (Supplementary Tables S6 and S7). A total of 25 adverse events (AEs) were reported across all three groups (low-dose *n* = 11, high-dose *n* = 7, and placebo *n* = 7), with no significant differences between groups. AEs were classified as mild (80%) or moderate (20%), and no serious AEs were reported. Seventeen AEs (68%) were considered treatment-related, with no clear dose-dependent pattern of treatment-related AEs. Most AEs (19 events, 76%) were transient and gastrointestinal in nature. Two participants (one placebo, one high-dose) withdrew due to AEs. These findings indicate overall good tolerability of the treatments and an acceptable safety profile in this population.

## 4. Discussion

The present randomised, double-blind, placebo-controlled trial evaluated the effects of MaolactinGI™ supplementation on GI symptoms, digestive-related quality of life, and gut microbiome composition in adults experiencing moderate upper GI discomfort. Over the eight-week intervention period, supplementation with MaolactinGI™ resulted in significant improvements in several domains of GI symptoms, particularly in the high-dose group.

The primary outcome measure, the GSRS, revealed significant improvements in abdominal pain, indigestion, and constipation in participants receiving the higher dose of MaolactinGI™ compared with placebo. These improvements were accompanied by a reduction in the overall GSRS score, suggesting a meaningful reduction in total symptom burden. The low-dose group demonstrated numerical improvements across several domains; however, these changes did not reach statistical significance compared to placebo, possibly suggesting the presence of a dose-response, with the high-dose group also showing a significant advantage over the low-dose group in the diarrhoea domain. Currently, there is no single standardised clinical dose established for colostrum in the general population. Commercially available supplements typically recommend around 500 mg^−1^ g/day for healthy adults, whereas clinical studies examining colostrum’s effects have used a much wider range of doses, from under 1 g/day up to 60–100 g/day [34,35]. There is very little research examining dose-response effects of colostrum specifically in gastrointestinal conditions, and further studies are needed to determine an effective dose range.

Improvements in digestion-related QoL as measured by DQLQ scores were also observed. Participants receiving the higher dose experienced significant reductions in DQLQ scores beginning as early as Week 2 and continuing through Week 8. Importantly, these clinical improvements in digestive-related quality of life occurred without influencing the bowel movement frequency or stool consistency. Measuring digestive-related quality of life in GI clinical trials is important as it captures the patient’s subjective experience by evaluating how the person’s physical, mental, emotional, and social well-being are affected by their GI symptoms [36]. GI conditions have been known to exert a profound psychological impact on sufferers, creating anxiety and distress, and negatively impacting social interactions [37]. Clinical improvement on symptom rating scales and other clinical measures do not always correlate with overall improvements in a person’s sense of well-being. In this study, the higher dose MaolactinGI™ produced both a rapid onset of benefit and durable improvement in digestion-related quality of life across the full 8-week study period, indicating that its benefits extended beyond physical changes.

A notable finding of this study was that the between-group analyses supported a high-dose effect on microbial richness. Although the overall Chao1 group × time interaction approached but did not reach statistical significance, the prespecified high-dose-versus-placebo contrast was significant after adjustment for multiple comparisons. Chao1 richness remained stable in the high-dose group while declining in the placebo group, with observed richness showing a similar numerical pattern. This suggests that high-dose MaolactinGI™ may help preserve microbial richness over time. LinDA also identified a high-dose-specific change in *Parasutterella* relative to placebo, although the lack of confirmation by ANCOM-BC2 indicates that individual taxonomic findings should be considered exploratory. PERMANOVA also did not identify a significant group × time interaction for overall community composition. Collectively, the between-group analyses suggest that the microbiome effects of MaolactinGI™ are encouraging and warrant confirmation in studies designed with microbiome outcomes as a primary endpoint.

A healthy gut is defined as not only being free of diagnosed GI disease, but also as the absence of any discomfort or signs of dysfunction [38]. Gut health is implicitly linked to the gut microbiome, as imbalances in the intestinal microbiota often lead to gut symptoms. The typical features of microbiota alteration, or dysbiosis, include reduced diversity and increased facultative anaerobes (such as *Enterobacteriaceae*). Dysbiosis commonly leads to inflammation and has been linked to the development of various gastrointestinal, metabolic, autoimmune, neoplastic, and neuropsychiatric disorders [39]. Increased microbial diversity is widely recognised as an indicator of a more stable and resilient gut ecosystem and has been associated with improved metabolic and immune regulation [40,41]. Participants receiving the higher dose of MaolactinGI™ demonstrated a significant increase in microbial diversity as measured by the Shannon diversity index, although a similar increase was observed in the placebo group. It should be noted that this reflects a within-group change from baseline. Taxonomic analysis revealed enrichment of beneficial commensal taxa in the high-dose group, including *Veillonella, Megasphaera*, and *Romboutsia*. These taxonomic findings were derived from within-group comparisons of baseline and Week 8 samples and were not identified in the between-group differential abundance analysis; they should therefore be considered hypothesis-generating and warrant confirmation in future studies. These bacterial genera are known to participate in metabolic cross-feeding pathways that generate short-chain fatty acids (SCFAs), including propionate and butyrate. SCFAs play an important role in maintaining intestinal epithelial integrity, regulating immune signalling, and supporting mucosal homeostasis [17]. If confirmed, the enrichment of these taxa would suggest that MaolactinGI™ may promote a gut environment supportive of beneficial microbial metabolism. The between-group preservation of richness would be consistent with a host-directed mechanism of action. Rather than directly introducing microbial species, bioactive proteins derived from colostrum may interact with epithelial receptors and mucosal immune pathways that shape the intestinal environment [12,13,14]. Through these interactions, the intervention may indirectly promote microbial diversity and beneficial bacterial communities.

Despite improvements in symptoms and preservation of microbial richness, inflammatory markers and intestinal permeability remained unchanged. This finding is likely explained by the relatively healthy status of the study population at baseline, as participants exhibited normal intestinal permeability and low baseline inflammation.

Overall, the intervention demonstrated a good safety profile [11,12,15]. All reported adverse events were non-serious and predominantly mild in severity. No clinically meaningful changes were observed in liver function markers or metabolic safety parameters.

The findings of this study can be compared to previous research related to bovine colostrum supplementation. A number of clinical studies related to bovine colostrum and its effects on gut health have previously been conducted. The systematic review conducted by Hajihashemi et al. (2024) on the effects of bovine colostrum supplementation on GI diseases included 22 clinical trials with 1427 patients [35]. The results showed that supplementation with bovine colostrum reduced the frequency of diarrhoea in 15 out of 20 interventional arms; however, most studies reported no change in its duration. Abdominal pain relief was notably improved in 4 out of 9 interventional arms. The study concluded that there is limited evidence on the effects of bovine colostrum on GI diseases, with mixed findings [35]. Oswal et al. (2025) [42] conducted a systematic review evaluating the effect of bovine colostrum on gut health in the paediatric population. Of the 8 included studies, some reported a positive effect on the duration and frequency of infectious diarrhoea in children, but the overall clinical benefits of bovine colostrum on the gut health of children remain inconclusive, owing to heterogeneous methodology and variations in the dose and form of colostrum used [42]. Dziewiecka et al. (2022) [43] conducted a systematic review on the influence of bovine colostrum supplementation on leaky gut syndrome in athletes. In the 9 clinical studies evaluated, colostrum was shown to be highly beneficial in improving gut permeability [43]. Bovine colostrum has also been shown to be useful in the treatment of inflammatory bowel disease, irritable bowel syndrome, and non-steroidal anti-inflammatory (NSAID)-induced enteropathy [44].

In the absence of a widely established instrument-specific minimal clinically important difference (MCID) or responder threshold for the GSRS [45], clinical relevance was assessed using the widely applied distribution-based convention that an effect size of approximately 0.5 standard deviations (SD) approximates the minimally important difference across health-related quality-of-life measures [46]. Effect sizes were calculated as the mean between-group difference in change from baseline at Week 8 divided by the baseline SD. Applying this to our data, the between-group effect sizes for total GSRS (ES ≈ 0.7) and each significantly improved domain (abdominal pain, ES ≈ 0.7; indigestion, ES ≈ 0.7; constipation, ES ≈ 0.6) meet or exceed this benchmark, supporting the clinical, and not merely statistical, relevance of the observed improvements.

One limitation of the study is that the study population consisted of generally healthy adults with moderate GI symptoms rather than individuals with diagnosed GI disorders. Future studies in populations with functional GI disorders or higher baseline symptom severity may provide further insight into the therapeutic potential of MaolactinGI™.

## 5. Conclusions

Supplementation with MaolactinGI™ at a dose of 500 mg/day over 8 weeks significantly improved upper GI symptoms and digestive-related quality of life, and preserved gut microbial richness relative to placebo in adults experiencing moderate GI discomfort. These findings support the potential of targeted colostrum-derived bioactive protein fractions as a novel nutritional strategy for supporting GI health and microbiome balance. The current study adds to the growing body of research on the health benefits of bovine colostrum supplementation.

## Figures and Tables

**Table 1:** Participant Baseline Characteristics.

Mean (SD)	Low-Dose (LD) (*n* = 29)	High-Dose (HD) (*n* = 29)	Placebo (P) (*n* = 25)	*t*-Test LD vs. P	*t*-Test HD vs. P	*t*-Test HD vs. LD
Age (years)	48.69 (13.66)	50.76 (13.68)	45.52 (15.17)	0.421	0.186	0.566
Height (m)	1.67 (0.08)	1.62 (0.08)	1.66 (0.09)	0.679	0.060	0.009
Weight (kg)	68.24 (12.83)	65.62 (18.11)	74.50 (13.03)	0.129	0.083	0.583
BMI	22.72 (5.79)	24.61 (5.30)	27.06 (3.72)	0.007	0.120	0.989
Waist (cm)	82.92 (11.20)	81.51 (14.78)	88.21 (12.54)	0.129	0.098	0.6998
Hips (cm)	102.98 (9.06)	97.92 (12.32)	103.76 (10.49)	0.782	0.084	0.095
Systolic BP (mmHg)	113.82 (19.32)	121.86 (16.90)	117.95 (12.08)	0.425	0.405	0.148
Diastolic BP (mmHg)	75.91 (10.88)	81.09 (10.35)	77.95 (7.27)	0.492	0.274	0.113
Pulse (BPM)	67.18 (10.05)	70.27 (9.19)	72.00 (11.65)	0.162	0.598	0.292

SD = Standard Deviation, BP = blood pressure.

**Table 2:** Gastrointestinal Symptom Rating Scale Scores.

Average (SD)	Low-Dose (*n* = 29)				High-Dose (*n* = 29)				Placebo (*n* = 25)			
Timepoint	BL	W2	W4	W8	BL	W2	W4	W8	BL	W2	W4	W8
Reflux	4.71 (2.76)	3.36 (2.13)	3.68 (2.40)	3.39 (2.20)	5.56 (2.52)	3.63 (1.96)	4.32 (2.01)	3.44 (1.28)	5.36 (2.80)	4.36 (3.02)	3.83 (2.08)	4.04 (2.36)
Abdominal Pain	7.93 (3.56)	6.93 (3.79)	5.71 (2.37)	5.61 (2.54)	7.78 (2.49)	5.89 (2.42)	6.04 (1.97)	4.81 (1.62)	7.36 (2.48)	6.18 (3.10)	5.91 (3.19)	5.83 (3.05)
Indigestion	13.21 (4.83)	11.18 (5.21)	9.68 (4.36)	10.07 (5.04)	12.63 (3.60)	9.63 (4.02)	9.16 (3.58)	7.81 (3.21)	13.18 (4.57)	10.95 (5.05)	9.87 (4.78)	10.35 (5.46)
Diarrhoea	5.57 (3.51)	5.75 (4.51)	5.64 (3.98)	5.71 (4.04)	6.78 (3.41)	6.15 (3.78)	6.16 (4.59)	5.04 (3.20)	7.14 (3.50)	6.91 (4.81)	5.57 (4.60)	5.48 (3.93)
Constipation	8.21 (4.75)	8.18 (4.71)	6.89 (4.57)	6.50 (3.62)	8.07 (3.96)	5.96 (2.81)	6.16 (2.49)	5.41 (2.56)	6.32 (3.06)	5.41 (2.65)	5.91 (3.16)	5.61 (3.54)
Total GSRS	7.93 (2.88)	7.08 (3.36)	6.32 (2.66)	6.26 (2.85)	8.16 (1.76)	6.25 (2.21)	6.37 (1.89)	5.30 (1.59)	7.87 (2.29)	6.76 (3.19)	6.22 (3.09)	6.26 (2.89)

SD = Standard Deviation, BL = Baseline.

**Table 3:** Quality of Life and Gastrointestinal Questionnaire Scores.

Average (SD)	Low-Dose (*n* = 29)				High-Dose (*n* = 29)				Placebo (*n* = 25)			
Timepoint	BL	2	4	8	BL	2	4	8	BL	2	4	8
Total DQLQ	2.27 (1.92)	1.69 (1.88)	1.65 (1.91)	1.48 (1.64)	2.78 (1.27)	1.48 (1.27)	1.41 (1.10)	1.13 (1.04)	2.21 (1.23)	2.23 (1.77)	1.64 (1.71)	1.58 (1.62)
Total GERD-Q	6.36 (2.45)	6.14 (2.24)	6.36 (2.30)	6.32 (2.07)	7.65 (2.28)	6.46 (1.42)	7.24 (2.03)	6.89 (1.31)	7.09 (1.97)	6.72 (1.91)	6.68 (1.36)	6.95 (2.24)
BSVAS-stomach feeling full	5.64 (2.92)	4.46 (3.31)	4.50 (3.07)	3.50 (2.80)	5.89 (2.47)	3.65 (2.95)	3.64 (2.55)	2.78 (2.22)	5.26 (2.54)	4.52 (2.91)	4.00 (2.62)	3.68 (3.03)
BSVAS-stomach distension	5.29 (3.13)	4.21 (3.34)	4.50 (3.12)	4.00 (2.97)	5.26 (3.06)	2.96 (2.58)	2.88 (2.70)	2.74 (2.99)	4.87 (2.78)	3.52 (2.93)	3.36 (2.66)	2.91 (2.99)
BSVAS-tight skin on stomach	3.93 (3.52)	3.82 (3.32)	3.68 (3.33)	3.11 (3.13)	3.70 (2.98)	2.58 (2.53)	3.00 (2.71)	1.96 (2.47)	2.78 (3.12)	2.29 (2.63)	1.86 (2.62)	1.86 (2.96)
Bristol stool shape	3.39 (1.31)	2.96 (1.61)	3.38 (1.63)	3.78 (1.40)	3.59 (1.28)	3.69 (1.12)	3.92 (1.15)	3.74 (1.40)	4.35 (1.50)	4.45 (1.65)	4.09 (1.28)	3.90 (1.48)
Number of bowel movements	8.43 (4.16)	8.15 (4.31)	8.62 (4.07)	8.74 (4.23)	8.93 (4.27)	9.12 (4.17)	9.88 (5.36)	9.22 (4.90)	9.00 (3.91)	8.55 (4.81)	8.87 (4.97)	7.86 (3.53)

SD = Standard Deviation, BL = baseline.

## Data Availability

Data are available from the authors on request.

## Supplementary Materials

The following supporting information can be downloaded at: https://www.mdpi.com/article/10.3390/nu18213561/s1, Table S1: Inclusion and Exclusion Criteria; Table S2: Participant Characteristics at Baseline and Week 8; Table S3: Inflammatory Markers; Table S4: Faecal Calprotectin ug/g; Table S5: Lactulose:Mannitol Ratio; Table S6: Liver Safety Markers; Table S7: Metabolic Safety Markers.

## Abbreviations

The following abbreviations are used in this manuscript:

BSVAS	Bloating Symptoms Visual Analogue Scale
DQLQ	Digestion-associated Quality of Life Questionnaire
GI	Gastrointestinal
GMP	Good manufacturing procedures
GSRS	Gastrointestinal Symptom Rating Scale
mITT	Modified intention-to-treat
QOL	Quality of life
SCFA	Short-chain fatty acids
