# Supplementary material for: Targeted Colostrum Bioactive Proteins Improve Gastrointestinal Symptoms and Modulate the Gut Microbiome: A Randomised Double-Blind Placebo-Controlled Trial
Source: Nutrients. 2026 Oct 28;18(21):3561. doi: 10.3390/nu18213561 (PMC13764744; doi:10.3390/nu18213561)
Supplement: Supplementary file 1 [file nutrients-18-03561-s001.zip › nutrients-4430303-supplementary.pdf]

Supplementary Data

Table S1. Inclusion and Exclusion Criteria

Inclusion criteria
• Adults 18 years and over<br>• Generally healthy<br>• Able to provide informed consent<br>• Agree not to participate in another clinical trial while enrolled in this trial<br>• Females using a prescribed form of birth control (e.g. oral contraceptive)<br>• Experiencing moderate GI disturbances of the upper GI tract - 1 or multiple symptoms (reflux, heartburn, regurgitation, nausea, bloating, abdominal pain) at least once a week for at least 3 months.<br>• Normal dietary habits (no FODMAP diet, elimination diet, vegan diet, etc) with a minimum 2-month period of self-reported dietary stability.<br>• Agree to not change current diet or exercise regime during entire study period<br>• Agree to not use any dietary supplements for gut health or digestive enzymes during the study period
Exclusion criteria
• Unstable or serious illness (e.g. serious mood disorders such as depression or bipolar disorder, neurological disorders such as MS, kidney disease, liver disease, heart conditions, diabetes, thyroid gland dysfunction)[1]<br>• People with a past or current history of GIT conditions e.g. inflammatory bowel disease, celiac disease or cystic fibrosis as well as gastrointestinal tract surgery<br>• Current malignancy (excluding BCC) or chemotherapy or radiotherapy treatment for malignancy within the previous 2 years<br>• Currently taking any proton pump inhibitors [e.g., Somac (pantoprazole), Pariet (rabeprazole), Losec (omeprazole)] or any anticoagulation or antiplatelet medications [e.g., Coumadin (Warfarin), Heparin, Dalteparin, Enoxaparin, dabigatran (Pradaxa), rivaroxaban (Xarelto), apixaban (Eliquis), edoxaban (Savaysa), betrixaban (Bevyxxa), clopidogrel (Plavix), prasugrel (Effient), ticagrelor (Brilinta), cilostazol (Pletal) and dipyridamole (Attia, Ofcram, Persantin, Persantin Retard, Trolactin)] including low dose aspirin (acetylsalicylic acid).<br>• Active smokers, nicotine use or drug (prescription or illegal substances) abuse<br>• Allergic to any of the ingredients in active or placebo formula<br>• Pregnant or lactating woman or women trying to conceive<br>• Any condition which in the opinion of the investigator makes the participant unsuitable for inclusion<br>• Currently participating in any other clinical trial<br><br>[1]An unstable illness is any illness that is currently not being treated with a stable dose of medication or is fluctuating in severity. A serious illness is a condition that carries a risk of mortality, negatively impacts quality of life and daily function and/or is burdensome in symptoms and/or treatments. Any potential participants will be advised at the time of screening of their inability to participate in the trial if they fail to meet any of the stated conditions.

Table S2. Participant Characteristics at Baseline and Week 8

	Baseline			8 Weeks		
Group<br>Mean (SD)	Low Dose<br>(n=29)	High Dose<br>(n=29)	Placebo<br>(n=25)	Low Dose<br>(n=29)	High Dose<br>(n=29)	Placebo<br>(n=25)
Age (years)	48.69 (13.66)	50.76 (13.68)	45.52 (15.17)			
Height (m)	1.67 (0.08)	1.62 (0.08)	1.66 (0.09)	1.67 (0.08)	1.62 (0.08)	1.66 (0.09)
Weight (kg)	68.24 (12.83)	65.62 (18.11)	74.50 (13.03)	68.65 (13.16)	65.03 (18.36)	74.44 (13.53)
BMI	22.72 (5.79)	24.61 (5.30)	27.06 (3.72)	24.51 (3.79)	24.49 (5.43)	26.99 (3.93)
Waist circumference (cm)	82.92 (11.20)	81.52 (14.78)	88.21 (12.54)	83.55 (12.15)	79.72 (13.83)	86.49 (11.61)
Hips circumference (cm)	102.98 (9.06)	97.92 (12.32)	103.76 (10.49)	103.13 (10.01)	97.11 (10.31)	107.00 (8.85)
Blood pressure - Systolic	113.82 (19.32)	121.86 (16.90)	117.95 (12.08)	112.82 (14.26)	117.43 (17.81)	118.2 (11.31)
Blood pressure - Diastolic	75.91 (10.88)	81.09 (10.35)	77.95 (7.27)	76.09 (10.61)	76.24 (10.19)	78.06 (7.77)
Pulse	67.18 (10.05)	70.27 (9.19)	72.00 (11.65)	66.91 (9.19)	69.33 (11.24)	73.12 (15.29)

SD = standard deviation

Table S3. Inflammatory Markers

Average (SD)	NF-kB (%)		IL-1b (pg/mL)<br>Ref: 0.5-5 pg/mL		IL-6 (pg/mL)<br>Ref:0-43.5 pg/mL		IL-8 (pg/mL)<br>Ref: <57.8 pg/mL		MIP-1a (pg/mL)<br>Ref: <10 pg/mL		TNF-a (pg/mL)<br><7.2 pg/mL		CRP (mg/L)<br><0.4	
	Baseline	Final	Baseline	Final	Baseline	Final	Baseline	Final	Baseline	Final	Baseline	Final	Baseline	Final

Low Dose	0.66 (0.98)	0.71 (0.99)	1.58 (1.21)	1.59 (1.26)	7.27 (19.29)	7.16 (18.61)	14.00 (18.64)	14.34 (18.84)	9.36 (7.78)	8.25 (8.81)	6.60 (2.65)	6.88 (3.65)	2.58 (2.30)	2.67 (2.65)
High Dose	1.33 (2.28)	1.89 (4.22)	1.70 (1.37)	1.73 (1.38)	7.94 (13.14)	7.82 (13.69)	14.95 (17.29)	14.06 (14.45)	10.02 (13.73)	10.46 (11.99)	6.92 (3.48)	6.44 (2.97)	2.41 (2.65)	3.72 (3.44)
Placebo	7.03 (22.38)	6.72 (22.27)	1.75 (1.36)	1.71 (1.45)	8.31 (11.03)	8.00 (10.88)	16.51 (14.35)	15.40 (14.96)	9.41 (7.76)	11.80 (16.06)	6.86 (2.04)	7.04 (3.41)	4.42 (3.78)	3.61 (2.89)

SD = standard deviation

Table S4. Faecal Calprotectin ug/g

Average (SD)	Baseline	Week 8
Low Dose	3.71 (5.69)	3.54 (4.67)
High Dose	2.30 (1.36)	3.48 (6.17)
Placebo	2.43 (2.34)	1.86 (1.25)

SD = standard deviation

Table S5. Lactulose:Mannitol Ratio

	Baseline	Week 8
Low Dose	0.05 (0.10)	0.05 (0.09)
High Dose	0.04 (0.06)	0.05 (0.06)
Placebo	0.05 (0.08)	0.02 (0.03)

Table S6. Liver Safety Markers

Average (SD)	ALT (U/L)	AST (U/L)	GGT (U/L)

	Baseline	Week 8	Baseline	Week 8	Baseline	Week 8
Low Dose	17.98 (8.18)	19.11 (10.02)	23.11 (3.74)	25.25 (7.61)	19.29 (18.58)	20.86 (18.26)
High Dose	20.03 (10.30)	26.68 (21.14)	24.35 (7.62)	29.75 (18.00)	19.67 (9.52)	22.53 (13.36)
Placebo	16.56 (4.84)	17.89 (10.03)	24.94 (9.61)	24.12 (12.58)	21.53 (18.57)	21.00 (19.41)

SD = standard deviation

Table S7. Metabolic Safety Markers

Average (SD)	Albumin (g/L)		Cholesterol (mmol/L)		Creatinine (umol/L)		Glucose (mmol/L)		Triglycerides (mmol/L)		T Protein (g/L)	
	Baseline	Week 8	Baseline	Week 8	Baseline	Week 8	Baseline	Week 8	Baseline	Week 8	Baseline	Week 8
Low Dose	43.77 (1.55)	44.06 (2.27)	5.52 (1.43)	5.42 (1.23)	67.51 (14.04)	66.78 (11.96)	5.41 (1.04)	5.43 (1.27)	0.80 (0.42)	0.85 (0.51)	63.55 (3.81)	64.45 (4.06)
High Dose	42.84 (2.00)	43.85 (2.10)	5.30 (0.84)	5.42 (0.99)	68.39 (12.83)	67.93 (14.41)	5.35 (0.51)	5.25 (0.53)	0.89 (0.36)	0.92 (0.43)	63.75 (5.02)	64.47 (4.59)
Placebo	43.14 (2.16)	43.23 (2.84)	5.47 (0.87)	5.68 (0.89)	69.32 (19.05)	68.45 (19.52)	5.17 (0.47)	5.03 (0.32)	0.95 (0.36)	1.02 (0.62)	64.46 (3.81)	64.68 (5.10)

SD = standard deviation
